# Retraction: Ling, W. et al. Evaluation of Anti-Obesity Activity, Acute Toxicity, and Subacute Toxicity of Probiotic Dark Tea. *Biomolecules* 2018, *8*(4), 99

**DOI:** 10.3390/biom9030085

**Published:** 2019-03-04

**Authors:** Biomolecules Editorial Office

**Affiliations:** St. Alban-Anlage 66, 4052 Basel, Switzerland; biomolecules@mdpi.com

The *Biomolecules* Editorial Office has been made aware that the published paper [1] was previously published in chinese in *China Tea Processing* by the same authors [2]. In order to preserve academic integrity, the title paper [1] will be marked as retracted. We apologize to the readership of *Biomolecules* for any inconvenience caused. The decision to retract has been made in cooperation with the authors of the article [1].

MDPI is a member of the Committee on Publication Ethics and takes the responsibility to enforce strict ethical policies and standards very seriously. To ensure the addition of only high quality scientific works to the field of scholarly publication, [1] is retracted and shall be marked accordingly. 
